# A De Novo Mutation (c.2423A>G) in *SAMD9* Causing MIRAGE Syndrome With Intrauterine Growth Retardation and Renal Hypoplasia in a Chinese Family

**DOI:** 10.1155/humu/9470286

**Published:** 2026-04-17

**Authors:** Yuxin Huang, Jiahui Fu, Zhongzhi Gan, Fu Xiong, Honglei Zhu, Fang Yang

**Affiliations:** ^1^ Department of Gynaecology and Obstetrics, Zhujiang Hospital, Southern Medical University, Guangzhou, China, fimmu.com; ^2^ Department of Fetal Medicine and Prenatal Diagnosis, Zhujiang Hospital, Southern Medical University, Guangzhou, China, fimmu.com; ^3^ Department of Medical Genetics, School of Basic Medical Sciences, Southern Medical University, Guangzhou, China, fimmu.com

**Keywords:** de novo mutations, intrauterine growth retardation, MIRAGE syndrome, renal hypoplasia, *SAMD9*

## Abstract

**Background and Aims:**

MIRAGE syndrome is an autosomal‐dominant genetic disease primarily caused by a de novo mutation in the gene SAMD9 gene. This study is aimed at investigating the pathogenesis of MIRAGE syndrome through a Chinese case exhibiting intrauterine growth retardation and renal hypoplasia.

**Methods:**

We performed clinical exome sequencing to identify the pathogenic loci in the family. Further functional studies were conducted to understand the impact of the identified mutation.

**Results:**

We identified a de novo mutation in SAMD9 that causes MIRAGE syndrome: c.2423A>G p.(Tyr808Cys). This mutation was associated with a novel phenotypic combination of intrauterine growth retardation and renal hypoplasia in a fetus. In vitro functional experiments demonstrated that the SAMD9 mutation reduced its levels of mRNA and protein.

**Conclusion:**

This study expands the pathogenic mutation spectrum of MIRAGE syndrome and provides new insights into its pathogenic mechanism. The identified mutation in SAMD9 provides a potential target for understanding and treating this complex disease.

## 1. Introduction

Intrauterine growth retardation (IUGR) is diagnosed when a fetus does not attain its intrauterine growth potential; it usually manifests as an ultrasonographically estimated fetal weight (EFW) or an abdominal circumference (AC) below the 10th percentile for gestational age [1, 2]. Placental agenesis is the principal pathogenic factor, but genetic causes must also be considered [3]. IUGR is associated with several rare genetic defects, including IMAGe syndrome (caused by a CDKN1C mutation) and MIRAGE syndrome (caused by mutation in the gene [*SAMD9*] encoding sterile alpha motif domain‐containing protein‐9) [4, 5]. Therefore, when a fetus develops IUGR, physicians should consider rare genetic diseases.

MIRAGE syndrome (OMIM #617053) is a genetic defect caused principally by a heterozygous *SAMD9* gain‐of‐function (GOF) mutation; the gene lies on the long arm of Chromosome 7 [6]. The condition can trigger multisystem disorders, including myelodysplasia, infection, growth restriction, adrenal hypoplasia, an abnormal genital phenotype, and enteropathy [7]. Many cases have been reported; the clinical phenotypes are continuously expanding and now include placental agenesis, postnatal growth retardation, and several endocrine disorders [8]. Male neonates with MIRAGE syndrome exhibit abnormal genital development and even complete, female external genitalia. However, the symptoms of female patients are mild; only ovarian hypoplasia has been reported [9]. Most cases of MIRAGE syndrome are closely linked to primary adrenal insufficiency. However, we found that some such patients evidenced only mild adrenal insufficiency or even normal adrenal cortex function [10, 11].

*SAMD9* is a causative gene of MIRAGE syndrome. GOF mutations increase the (negative) effects of *SAMD9* on cellular proliferation [12]. In this study, we investigated a Chinese family with MIRAGE syndrome caused by a de novo mutation, c.2423A>G. We investigated the clinical phenotypes and the functions of the family, and we shed light on the pathogenesis of MIRAGE syndrome.

## 2. Materials and Methods

### 2.1. Patients

This study was approved by the Ethics Committee of Zhujiang Hospital (an affiliate of Southern Medical University, Guangdong, China). Written informed consent was obtained from all participants or their guardians. Cord blood/peripheral blood samples were obtained from all participants (one affected individual [II‐4] and two normal individuals [I‐1 and I‐2]). Clinical data and necropsy reports on the proband were collected (Figure 1).

### 2.2. Mutation Screening

A standard phenol/chloroform extraction method was used to obtain genomic DNA from cord/peripheral blood. Clinical exome sequencing of three individuals (two normal individuals, I‐1 and I‐2, and one affected individual, II‐4) (Figure 2a) was performed in collaboration with Amcarelab (Guangzhou, China). Primer‐BLAST (https://www.ncbi.nlm.nih.gov/tools/primer-blast/index.cgi; LI NK_LO C=BlastHome) software was used to design polymerase chain reaction (PCR) primers for exome sequencing of the genes. The PCR products were purified via agarose gel electrophoresis and sent to Sangon Biotech (Shanghai, China) for sequencing.

### 2.3. Bioinformatics

The three‐dimensional (3D) protein structures of wild‐type and mutant SAMD9 proteins were predicted using Iterative Threading ASSEmbly Refinement (I‐TASSER, https://zhanggroup.org/I-TASSER/) software. Polymorphism Phenotyping v2 (Polyphen‐2, http://genetics.bwh.harvard.edu/pph2/) software was used to predict conservation among species.

### 2.4. Plasmid Construction and Mutagenesis

Total RNA was isolated from peripheral blood lymphocytes employing RNAex Pro Reagent (Accurate Biology, Hunan, China). First‐strand cDNA was synthesized using a HiScript II First Strand cDNA Synthesis Kit (Vazyme, Jiangsu, China) according to the manufacturer’s instructions. The full‐length *SAMD9* coding sequence was amplified using primers containing *Xho*I and *Kpn*I restriction enzyme sites (forward primer 5 ^′^‐CCGCTCGAGATGGCAAAGCAACTTAACCTTC; reverse primer 5 ^′^‐CGGGGTACCGTAACAATTTCAATGTCATAAGCAAGT). The PCR products were cloned into the pEGFP‐N1 vector after double digestion with *XhoI* and *KpnI* (New England Biolabs, Beijing, China). The mutation was introduced into the wild‐type *SAMD9* plasmid via PCR‐based site‐directed mutagenesis (forward primer 5 ^′^‐AACAAGATAATGTCTGTCTTCTGCAGTACTC; reverse primer 5 ^′^‐GAGTACTGCAGAAGACAGACATTATCTTGTT). Recombinant plasmids were purified using an EndoFree Mini Plasmid Kit II (Tiangen, Beijing, China) and sequenced to ensure correctness.

### 2.5. RNA Analysis

Human embryonic kidney (HEK) 293T cells were cultured in Dulbecco’s Modified Eagle’s Medium (DMEM; Gibco, New York, United States) supplemented with 10% (*v*/*v*) fetal bovine serum. pEGFP‐N1 or other recombinant plasmids (1 *μ*g) were transfected into HEK293T cells at 70% confluence using PEI (Sigma‐Aldrich, St. Louis, Missouri, United States) according to the manufacturer’s instructions. Twenty‐four hours after transfection, total RNA was isolated employing RNAex Pro Reagent (Accurate Biology, Hunan, China) and reverse‐transcribed into cDNA using HiScript II Q RT SuperMix for qPCR (Vazyme, Jiangsu, China). Real‐time PCR was used to measure the relative levels of mRNAs transcribed from *SAMD9* and *SAMD9L* (using Power Green qPCR Mix) (GDSBio, Guangzhou, China). The *SAMD9* amplification primers were as follows: forward 5 ^′^‐ATGGCAAAGCAACTTAACCTTCC; and reverse 5 ^′^‐CCATTCACGTCTTGTTCAGTCA. The *SAMD9L* amplification primers were as follows: forward 5 ^′^‐ATTCCAAGCAACGGGATGTAG; and reverse 5 ^′^‐AGTCTCGGTTTCCTATGAGAAGT. *GAPDH* served as the reference gene for normalization of *SAMD9* and *SAMD9L* expression. The *GAPDH* primers were as follows: forward 5 ^′^‐GCACCGTCAAGGCTGAGAAC; and reverse 5 ^′^‐TGGTGAAGACGCCAGTGGA. Gene expression levels were calculated using the (2−*ΔΔ*CT) method. The transfection and real‐time PCR assays were repeated three times.

### 2.6. Western Blotting

To analyze the differences in protein expression between wild‐type and mutant *SAMD9*, HEK293T cells were placed in the wells of a six‐well dish and transfected with the pEGFP‐N1, pEGFP‐N1‐*cSAMD9*, or pEGFP‐N1‐*ΔcSAMD9* plasmid. Cells were harvested 48 h after transfection and homogenized in RIPA buffer (Beyotime, Shanghai, China) supplemented with 1% (*w*/*v*) phenylmethanesulfonyl fluoride (PMSF; Solarbio, Beijing, China) (to prevent protein degradation). The blots were incubated with mouse anti‐GFP (1:3000; Proteintech, Rosemont, Illinois, United States) overnight at 4°C and then with goat anti‐mouse IgG‐HRP (1:5000; Proteintech) at room temperature for 1 h. Bands were detected using the Immobilon Western Chemiluminescent HRP substrate (Thermo Fisher Scientific, Waltham, Massachusetts, United States).

### 2.7. Subcellular Localization

HEK293T cells were cultured in DMEM supplemented with 10% (*v*/*v*) fetal bovine serum at 37°C under 5% (*v*/*v*) CO_2_. pEGFP‐N1, pEGFP‐N1‐*SAMD9*, or pEGFP‐N1‐*ΔcSAMD9* was transfected into cells with the aid of PEI (Sigma‐Aldrich). After 48 h, the cells were washed three times with phosphate‐buffered saline (PBS; Gibco) and fixed for 30 min in 4% (*v*/*v*) paraformaldehyde (Leagene Biotechnology, Beijing, China). The paraformaldehyde was then discarded, and the samples were washed three times with PBS. To observe the subcellular locations of the SAMD9 proteins, nuclei were stained with 4 ^′^,6‐diamidino‐2‐phenylindole (DAPI; Sigma‐Aldrich). A confocal fluorescence microscope (LSM 880; Carl Zeiss AG, Jena, Germany) was then used to image the cells.

### 2.8. Cell Proliferation Assay

A CCK‐8 assay was performed to assess the effect of the mutation on cell proliferation. According to the protocol, transfected HEK293T cells were seeded into a 96‐well plate at a density of 1000 per well. During incubation for 5 days, 10 *μ*L of CCK‐8 solution (Yeason Biotechnology, Shanghai, China) was added to the wells daily, and the plate was incubated for 2 h. Next, the optical density (OD) at 450 nm was measured using a microplate reader (Victor X5, PerkinElmer).

## 3. Results

### 3.1. Clinical Phenotype

The proband was a fetus born to healthy nonconsanguineous parents. Her mother had earlier suffered an embryonic development arrest and two miscarriages. The family history was unremarkable. The fetus was referred to Zhujiang Hospital (Guangzhou, China) for further examination given the abnormal amniotic fluid volume. A four‐dimensional ultrasound performed at 24 + 5 weeks of gestation revealed IUGR (a size appropriate for 21–22 weeks of gestation), oligohydramnios, a single umbilical artery, and bilateral renal hypoplasia (Figures 1a, 1b, and 1c). After discussion with the parents and receipt of informed consent, labor was induced with Rivanol. A dead female fetus 3700 g in weight was delivered. The umbilical cord had been wrapped around the neck for 2 weeks and the ankle for 1 week. The autopsy report stated that the fetus was 32.8 cm long with dark red skin, a head circumference of 15.5 cm, and scalp edema, but no surface deformity (Figure 1g). Microscopic evaluation demonstrated bilateral renal hypoplasia with concomitant glomerular and tubular atrophy. Histopathological examination of the placenta demonstrated focal calcifications accompanied by neutrophilic infiltrates, without otherwise abnormalities (Figures 1d, 1e, and 1f).

Figure 1Clinical details of the proband. (a–c) Four‐dimensional ultrasound imaging performed at 24 + 5 weeks of gestation revealed bilateral renal hypoplasia (with volumes below the expected range for gestational age) and a single umbilical artery. (d–f) Histopathological examination of the placenta demonstrated focal calcifications accompanied by neutrophilic infiltrates, without otherwise abnormalities. (g) Autopsy data: The proband was 32.8 cm in length and had dark red skin, a head circumference of 15.5 cm, and scalp edema.

**Figure:**
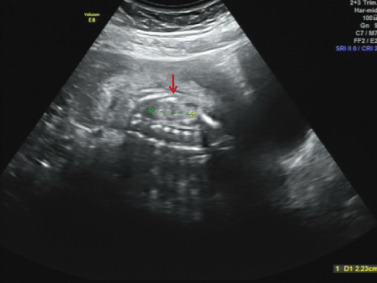
(a)

**Figure:**
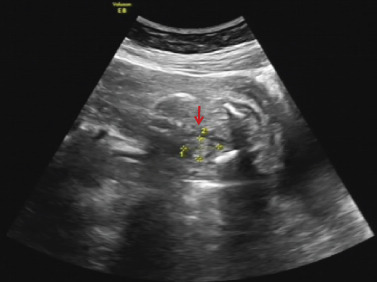
(b)

**Figure:**
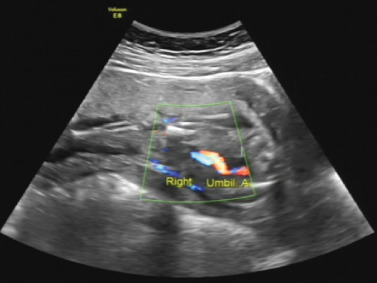
(c)

**Figure:**
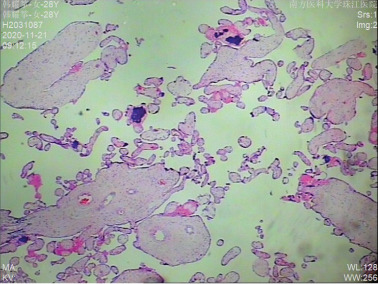
(d)

**Figure:**
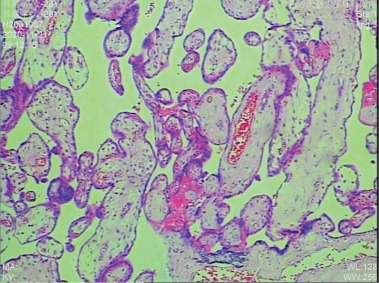
(e)

**Figure:**
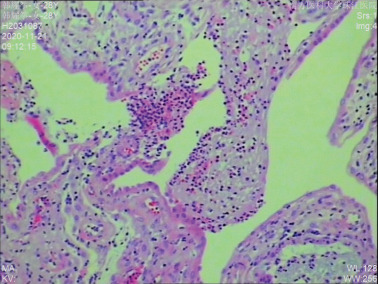
(f)

**Figure:**
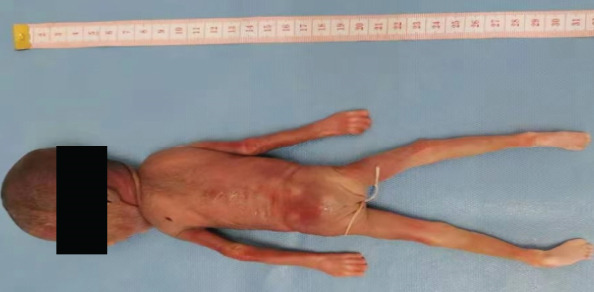
(g)

### 3.2. Mutational Analysis

To identify the pathogenic mutation, clinical exome sequencing was performed on the proband (II‐4) and her parents (I‐1 and I‐2) (Figure 2a). The proband exhibited a heterozygous mutation in *SAMD9* (NM_001193307:c.2423A>G(p.Y808 C)), which was absent in the parents, thus may be a de novo mutation. The mutation was confirmed via Sanger sequencing and cosegregation analyses of the proband and her unaffected family members (Figure 2b). The mutation has not been reported in any clinical case and is not in the reference population database. According to ACMG/AMP guidelines incorporating the updated ClinGen Sequence Variant Interpretation (SVI) recommendations, this variant meets PS2 (strong), PM2_supporting (supporting), and PP4 (supporting) criteria and can therefore be classified as likely pathogenic. The *SAMD9* mutation Y808 lay in an evolutionarily conserved region; the tyrosine at this site is consistent across species (Figure 2d). I‐TASSER indicated that the *SAMD9* c.2423A>G mutation changed the protein’s tertiary structure, principally parts of the alpha‐helix and the random coil (Figure 2c).

Figure 2(a) Pedigree of the family. Males are marked with squares, females with circles, and fetuses (of unknown sex) with triangles. The arrow indicates the proband and the black symbols affected individuals. (b) Sanger sequencing data. Only II‐4 carried a heterozygous mutation in *SAMD9* (c.2423A>G). (c) The 3D structure of the mutated SAMD9 differed from that of the wild‐type protein, as revealed by I‐TASSER. (d) Conservation analysis of the abnormal variation (Polyphen‐2 data). Amino acid 808 of SAMD9 was highly conserved among different species.

**Figure:**
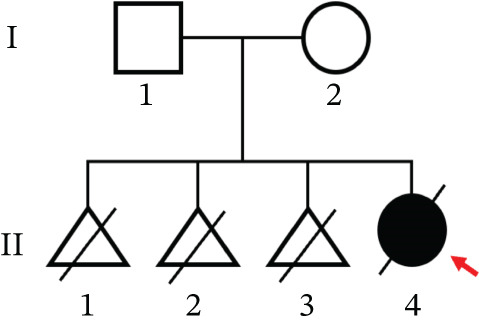
(a)

**Figure:**
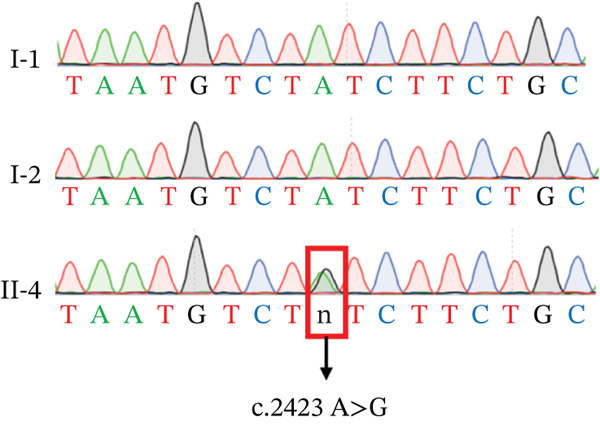
(b)

**Figure:**
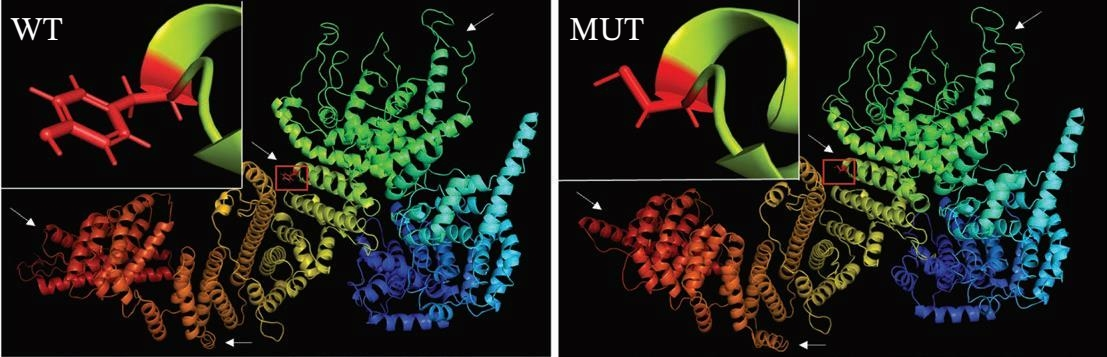
(c)

**Figure:**
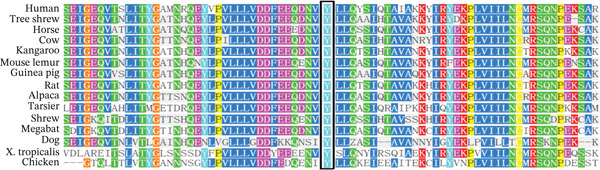
(d)

### 3.3. Functional Analysis

The functional effects of the *SAMD9* mutation were analyzed in HEK293T cells. As shown in Figures 3a, 3b, and 3c, the levels of encoding mRNAs and proteins differed significantly between cells transfected with pEGFP‐N1, pEGFP‐N1‐*SAMD9*, and pEGFP‐N1‐*ΔcSAMD9*. The *SAMD9* mRNA and protein expression levels were reduced by nearly 50% compared with the wild type. However, the subcellular localizations did not differ; both proteins were in the cytoplasm (Figure 3d). CCK‐8 assay showed that mutant *SAMD9* decreased the viability of HEK293T cells compared to those transfected with wild‐type *SAMD9* (Figure 3e). *SAMD9L* is a paralog of *SAMD9*; abnormal expression of the former can trigger multisystem diseases [13]. As shown in Figure 3f, compared to cells transfected with pEGFP‐N1‐*SAMD9*, the level of *SAMD9L* mRNA was reduced in cells transfected with pEGFP‐N1‐*ΔcSAMD9*.

Figure 3Effect of mutation on SAMD9 function. (a) The levels of *SAMD9* mRNAs in HEK293T cells. The mRNA levels of the wild‐type and mutant sequences differed significantly ( ^∗∗∗∗^ *p* < 0.0001). (b) Western blotting for SAMD9. (c) The protein expression levels in HEK293T cells. The mutant SAMD9 protein was expressed at a lower level than the wild‐type protein ( ^∗∗∗∗^ *p* < 0.0001). (d) Subcellular localizations of wild‐type and mutant SAMD9 in HEK293T cells. Both proteins lay principally in the cytoplasm. Confocal images of EGFP (green), DAPI nuclear staining (blue), and the merged signals. (e) Effect of mutant SAMD9 on the proliferation ability of HEK293T cells as determined by the CCK‐8 assay. (f) The mRNA expression levels of SAMD9L in HEK293T cells. The mRNA levels of the wild‐type and mutant gene differed significantly ( ^∗∗∗^ *p* < 0.001).

**Figure:**
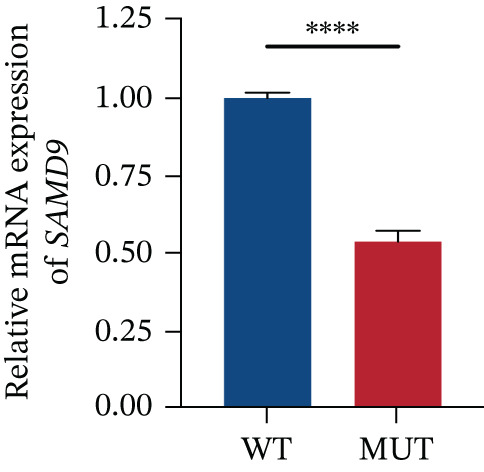
(a)

**Figure:**
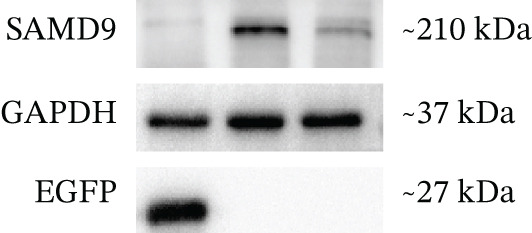
(b)

**Figure:**
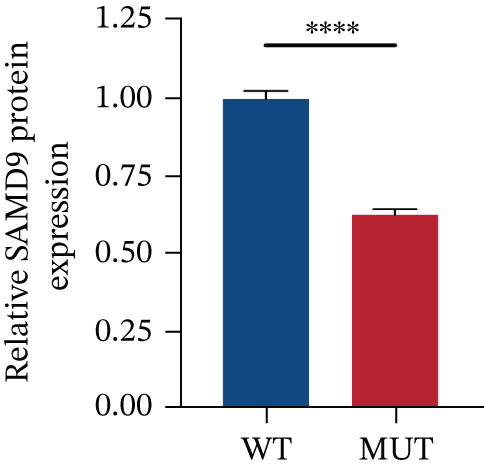
(c)

**Figure:**
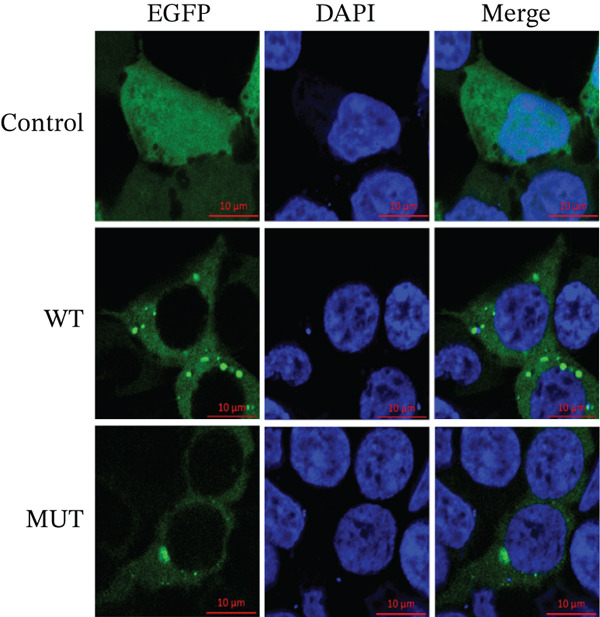
(d)

**Figure:**
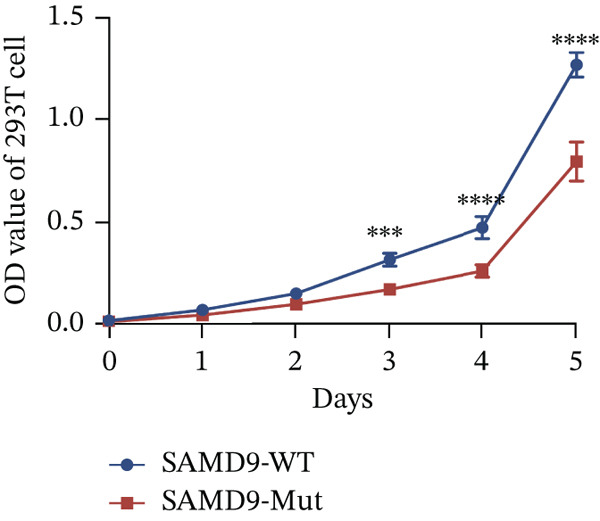
(e)

**Figure:**
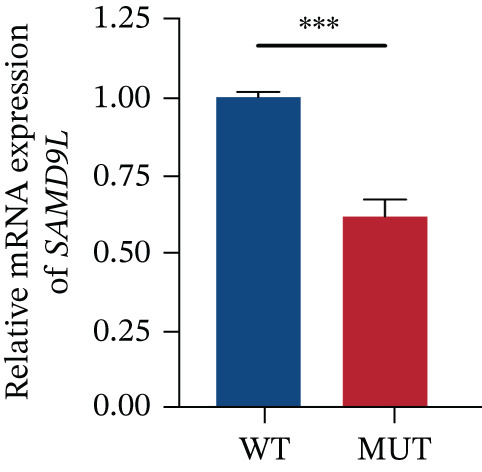
(f)

## 4. Discussion

We identified a novel phenotypic combination of fetal MIRAGE syndrome, IUGR, and renal hypoplasia, caused by a novel heterozygous mutation in *SAMD9* (c.2423A>G p.(Tyr808Cys)). We performed functional studies to confirm the pathogenicity of the mutation.

MIRAGE syndrome is an autosomal‐dominant disorder typically caused by de novo pathogenic mutations [14]. MIRAGE syndrome caused by *SAMD9* mutations exhibits a variety of different phenotypes, among which endocrine features (primary adrenal insufficiency and gonadal dysgenesis) are the initial core components. However, IUGR and intrauterine death are the main manifestations during the fetal period; other organ disorders gradually emerge in childhood [8, 15]. In this study, the proband exhibited a novel phenotypic combination of MIRAGE syndrome, thus IUGR and renal hypoplasia. The MIRAGE syndrome phenotype can be gradually reversed (in a natural way), for example, by progressive loss of the mutant allele (via Monosomy 7 or 7q) or a somatic reversion of the loss‐of‐function mutation [16]. One MIRAGE syndrome patient of Karyotype 46, XY presented with IUGR, disorders of sex development, thrombocytopenia, and necrotizing enterocolitis. These phenotypes became rapidly corrected on about Day 20; the patient is now a healthy girl [17]. Unfortunately, the fetus in our study was induced at 25 weeks gestation (because the family requested it).

SAMD9 is a widely expressed innate immune response protein and tumor suppressor in humans. This protein is encoded by a single exon gene located on human chromosome 7q21. It contains an RNA‐nonbinding SAM domain and can undergo oligomerization with both SAM domain‐containing and SAM domain‐lacking proteins [18, 19]. Under physiological conditions, SAMD9 is activated by signals such as interferon and negatively regulates cell growth by inhibiting protein synthesis, among other pathways, in response to infection or cellular stress [20]. Deleterious mutations in the SAMD9 gene are known to cause normophosphatemic familial tumoral carcinosis (NFTC) and MIRAGE syndrome [21, 22]. In our study, the mutation (c.2423A>G) decreased SAMD9 mRNA expression and the protein level and the viability of HEK293T cells. Combined with the clinical phenotype, the de novo mutation results in a GOF. The core of GOF centers on enhancing protein function rather than increasing protein quantity. We propose two potential mechanisms that confer a GOF in SAMD9. The first is gain of structural domains [23]. Protein domains are evolutionarily conserved regions with independent functional properties. The structure–function relationship encoded in protein domains has been used for understanding the functional effects of disease‐related mutations [24]. The mutant protein (p.Y808 C) can acquire a new domain through various mechanisms, which may result in tighter binding of SAMD9 to its downstream targets or sustain the activation of its inhibitory enzymatic activity. This enhanced function ultimately strengthens the suppression of cell proliferation. The second mechanism involves dysregulation of negative control [25]. Normal protein activity is tightly regulated within the organism. In its resting state, the immunomodulatory protein SAMD9 remains autoinhibited by its own structure [26]. The mutation (c.2423A>G) disrupts essential ionic bonds, hydrogen bonds, or hydrophobic interactions between the regulatory and catalytic domains. This disruption leads to constitutive SAMD9 activation, which in turn enhances the inhibition of cell proliferation. According to I‐Mutant2.0 (https://folding. http://biofold.org/i-mutant/i-mutant2.0.html), the mutant protein is less stable than its wild‐type counterpart and is more readily recognized and degraded by the cellular surveillance system, resulting in reduced SAMD9 expression. Before its degradation, the aberrantly increased activity of the mutant protein may enhance the inhibition of cell proliferation.

*SAMD9L* is a paralog of *SAMD9*; the protein amino acid identity level is 58%^27^. The tumor suppressor *SAMD9L* plays key roles in cell proliferation and innate immunity [27].

*SAMD9L* mutations are associated principally with immunodeficiency and nervous system diseases; most *SAMD9* mutations cause MIRAGE syndrome [11, 28]. Both genes are present in most mammals; however, mice have only *SAMD9L* and cows only *SAMD9*, indicating that the two genes may (to some extent) be mutually complementary in terms of function [29, 30]. The mutation in the present study reduced the levels of both *SAMD9* and *SAMD9L* mRNAs, suggesting that any complementary effects disappear under pathological conditions. *SMAD9* mutation downregulates *SAMD9L* expression and aggravates disease progression. The regulatory interactions between the two genes thus require further study.

In conclusion, we identified a de novo mutation (c.2423A>G) in *SAMD9* associated with a new (combined) clinical phenotype of MIRAGE syndrome. Functional analysis was performed to elucidate the pathogenic mechanism. Our results expand the mutational spectrum of *SAMD9* in MIRAGE syndrome and shed light on the interaction between *SAMD9* and *SAMD9L*. However, further work is required.

## Author Contributions

All authors made substantial contributions to conception and design, acquisition of data, or analysis and interpretation of data. Yuxin Huang and Fang Yang took part in drafting the article or revising it critically for important intellectual content. Yuxin Huang and Jiahui Fu have contributed equally to this work.

## Funding

The study was funded by the Natural Science Foundation of Guangdong Province (2023A1515140149) and the Guangzhou Municipal Science and Technology Program Key Projects (2024B03J1036).

## Disclosure

All authors agreed to submit to the current journal. All authors gave final approval of the version to be published and agree to be accountable for all aspects of the work.

## Ethics Statement

The study was approved by the Ethics Committee of Zhujiang Hospital, Southern Medical University (Approval No. 2021‐KY‐119‐01), on November 1, 2021.

## Consent

Written informed consent was obtained from the patient for the publication of this report and any accompanying images.

## Conflicts of Interest

The authors declare no conflicts of interest.

## Data Availability

The data that support the findings of this study are available from the corresponding authors upon reasonable request.
